# Proportion of infants meeting the Australian 24-hour Movement Guidelines for the Early Years: data from the Melbourne InFANT Program

**DOI:** 10.1186/s12889-017-4856-9

**Published:** 2017-11-20

**Authors:** Kylie D. Hesketh, Katherine L. Downing, Karen Campbell, David Crawford, Jo Salmon, Jill A. Hnatiuk

**Affiliations:** Institute for Physical Activity and Nutrition (IPAN), School of Exercise and Nutrition Science, Deakin University, Geelong, Australia

**Keywords:** Early childhood, Movement guidelines, Physical activity, Sedentary behaviour, Sleep

## Abstract

**Background:**

Little information is available on the movement behaviours of infants, despite evidence that these are important for development. The release of new Australian 24-hour Movement Guidelines provides an opportunity to document the current state of movement behaviours in infants relative to these guidelines. The aim of this study was to report the prevalence of 4 month old Australian infants meeting the 24-hour Movement Guidelines, individually, and in combination, and to describe associations with individual characteristics.

**Methods:**

Maternal report baseline data from the Melbourne Infant Feeding, Activity and Nutrition Trial Program were used to determine prevalence of infants meeting physical activity (30 min of tummy time per day), sedentary behaviour (no more than 1 h at a time kept restrained; zero screen time), and sleep guidelines (14–17 h for 0–3 month olds or 12–16 h for 4–11 month olds). Prevalence of infants meeting combined guidelines was also described. The odds of meeting guidelines based on infant and family characteristics was determined.

**Results:**

Data are reported for 455 infants with a mean age of 3.6 months (SD = 1.0). The proportion of infants meeting each of the guidelines was 29.7% for tummy time, 56.9% for kept restrained, 27.9% for screen time, 58.7% for sleep and 3.5% for the combined guidelines (i.e. meeting all four guidelines). A significantly higher proportion of girls than boys met the screen time guideline (32.5% versus 24.0%, *p* = 0.04) and the combined guidelines (5.7% versus 1.6%, *p* = 0.01). Few associations were observed between infant and family characteristics and proportion of infants meeting individual guidelines.

**Conclusions:**

Very few infants met all of the guidelines contained in the new Australian 24-hour Movement Guidelines suggesting there is much room for improvement in movement behaviours from early life. Fewer infants met the tummy time and screen time guidelines hence these appear to be the behaviours requiring most attention. Parents and others providing care to infants require support and strategies to assist them in adhering to the guidelines to ensure optimal health and development for the youngest in our population.

## Background

In recent years, there has been increasing interest in the promotion of healthy behaviours from an early age, including physical activity, sedentary behaviour and sleep. This has largely come as a result of a growing body of evidence suggesting that these behaviours are important for children’s health. Recent systematic reviews have shown that greater amounts of physical activity, limited time spent in sedentary behaviours, and optimal sleep duration are independently associated with positive physical, psychosocial and cognitive health outcomes in early childhood [1–3]. Further, the combined effect of sufficient engagement in each of these behaviours appears to provide optimal outcomes for both children and youth [4, 5]. For these reasons, 24-h movement guidelines for the early years have recently been developed within Canada and Australia, with a focus on these health behaviours in three age groups: infants (birth – <1 year), toddlers (1–2 years) and preschool children (3–5 years) [6, 7].

Despite the recognised importance of promoting healthy behaviours from birth, most research conducted to date in the early childhood period has focused on children 2–5 years of age. Very little is collectively known about the proportion of children meeting physical activity, sedentary behaviour and sleep recommendations within the first few months of life. However such information is important for understanding the need for, and implementation of, public health programs in infancy.

For children under 1 year of age the Australian 24-hour Movement Guidelines for the Early Years recommend that children engage in:

1. Physical activity: Being physically active several times in a variety of ways, particularly through interactive floor-based play; more is better. For those not yet mobile, this includes at least 30 min of tummy time (time spent on the child’s stomach while awake) spread throughout the day while awake;
2. Sedentary behaviour: Not being restrained for more than 1 h at a time (e.g., in a stroller, car seat or high chair). Screen time is not recommended; and
3. Sleep: 14–17 h (0–3 months) and 12–16 h (4–11 months) of good quality sleep, including naps [7].

Previous studies on the prevalence of infants’ physical activity [8], sedentary time [9] and/or sleep [10, 11] have predominantly focused on the average duration of these behaviours, rather than the proportion of children meeting recommendations per se. Only a small body of literature reports on any aspect of the present 24-hour Movement Guidelines in the infant population and none consider multiple guidelines. Research from the USA found that 34% of 2 month old infants achieved 30 min daily tummy time [12], corresponding to the current guideline. Similarly, in two separate samples of 4 month old USA infants 23% [13] and 40% [14] were found to meet this same threshold for tummy time. Thus, based on current evidence, it appears that less than half of infants engage in sufficient tummy time to meet the guideline of 30 min per day.

The prevalence of 0–2 year olds meeting the screen time component of the sedentary behaviour guidelines has been investigated in a recent systematic review [9]. That review found that international estimates of meeting screen time guidelines (i.e., no screen time at all in infancy) range from 2% to 83%, varying by measure (i.e., screen exposure such as having television on in the background vs. direct viewing) and population group [9]. Moreover, all of the studies in that review measured only television viewing exposure, and not other screen behaviours, so the prevalence of young children meeting screen time guidelines that incorporates time spent with other screens or devices may in fact be lower than reported. No studies have documented the proportion meeting the restraint component of the sedentary behaviour guidelines, though the average duration of particular types of restraint behaviours (e.g., time in a car seat, stroller, etc.) have been previously described [15, 16].

Although little is known about the proportion of infants meeting sleep recommendations, two studies have been conducted examining guideline compliance in toddlers. One of those studies was conducted with a sample of 1–3 year olds in Italy and found that 66% of toddlers achieved the duration of sleep consistent with the new guidelines described above [17]. A study from the Special Supplemental Nutrition Program for Women, Infants, and Children in Puerto Rico reported that 48% of 29 month olds were achieving the duration of sleep recommended in the guidelines [18]. However, no studies were identified that considered all guidelines together.

Given the paucity of research within this field, there exists a need to better understand infants’ early compliance with current recommendations across all health behaviour domains, including physical activity, sedentary behaviour and sleep. The aim of this study was to report the prevalence of infants from an Australian sample that meet the 24-hour Movement Guidelines, individually, and in combination, and to describe associations with individual characteristics.

## Methods

### Sample

Data were from the baseline assessment of the Melbourne Infant Feeding, Activity and Nutrition Trial (InFANT) Program in 2008. This cluster-randomised controlled trial aimed to prevent obesity and obesity-promoting behaviours (spanning diet, physical activity and sedentary behaviours) during early childhood. The trial has been described in detail elsewhere [19, 20]. For the purposes of this paper, only details relating to recruitment and baseline assessments (prior to the intervention) are presented.

The Melbourne InFANT Program recruited from 14 local government areas (LGAs) randomly selected from all those within a 60 km radius of Deakin University’s Burwood campus, located in Melbourne, Victoria, Australia (population 4 million). Within participating LGAs, 50% of first-time parents’ groups (rounded to the next even number to allow 1:1 randomisation for the trial) were randomly approached for participation in the study (*n* = 62 groups). First-time parents’ groups are formed and facilitated by the universal Maternal and Child Health service within Victoria, Australia. They are predominantly attended by mothers and in this study all participants were mothers. Where a group declined or did not meet inclusion criteria (minimum of eight within a group consenting to participate, or six in low socioeconomic areas) the next group on the randomly generated list was approached.

Approval to conduct the Melbourne InFANT Program was granted by Deakin University’s Human Research Ethics Committee (EC 175–2007).

### Measures

For each of the infant behaviours assessed, corresponding to the 24-h Movement Guidelines, mothers reported the number of hours and minutes spent engaged in the behaviour on an average day via a written questionnaire, purposely developed for the study. Two-week test-retest reliability in a separate sample of 66 mothers with infants 1–5 months of age indicated variability in the reliability of reporting infant movement behaviours but that the majority of the items (6/10) demonstrated intra-class correlations (ICC) in the moderate to excellent range (i.e. ICC > 0.40) [21].

#### Infant physical activity

Physical activity was assessed as average daily tummy time (ICC = 0.25). Infants were classified as meeting the physical activity guideline if they were reported to have 30 min or more tummy time on an average day.

#### Infant sedentary behaviour

Sedentary behaviour was assessed as average daily television viewing (ICC = 0.69) and as daily time reported in six situations that restrict movement (bouncer or swing (ICC = 0.20), stroller or pram (ICC = 0.43), car seat or capsule (ICC = 0.50), high chair or other chair (ICC = 0.21), playpen (ICC = 0.27), and baby carrier or sling (ICC = 0.70)). Infants were classified as meeting the screen time guideline if mothers reported zero television viewing. They were classified as meeting the restraint guideline if they were reported to have 60 min or less in each of the six situations that restrict movement, on an average day.

#### Infant sleep

Sleep was assessed as the sum of average sleep duration at night (ICC = 0.70) and during the day (ICC = 0.76). Infants up to the age of 3.9 months were classified as meeting the sleep guideline if they had 14–17 h of sleep per day and infants aged 4 months and older were classified as meeting the sleep guideline if they had 12–16 h of sleep per day.

#### Combined guidelines

The number of guidelines that an individual infant met was summed. Infants were classified as meeting the combined guidelines if they met each of the individual guidelines (i.e., physical activity, screen time, restraint and sleep).

#### Family demographics and infant characteristics

Mothers reported their own and their infant’s date of birth, from which the age of both was calculated. Maternal height and prepregnancy weight were reported, from which prepregnancy body mass index (BMI) was calculated. Other demographics captured were mothers’ highest level of education, country of birth and the main language spoken at home.

Infants had their length and weight measured by trained research staff using a calibrated measuring mat (Seca 210, Seca Deutschland, Germany) and calibrated infant scales (Tanita 1582, Tokyo, Japan), respectively. BMI z-scores and weight-for-age percentiles were calculated from measured length and weight and based on exact age and sex using the World Health Organisation growth charts [22]. Infant temperamental ease was assessed by the single item “Compared with other children, I think my baby is:” [23] Response options were on a five point likert scale with “much easier than average” and “easier than average” combined for this study, “more difficult than average” and “much more difficult than average” combined, and “average” used as the referent category. Mothers also reported the amount of time per week (in hours) their infant was usually cared for by others.

### Analyses

For the purposes of the current study, participants who had complete data on all of the infant behaviours and the infant and family characteristics were included in analyses. Participants who were more than 6.99 months of age (*n* = 23; 14 aged 7–8 months and nine aged 9 months or older) were excluded to limit the developmental range of the sample. Frequencies were used to describe characteristics of the sample and to describe the proportion of infants (all and stratified by sex) meeting each of the separate and the combined guidelines, respectively. Differences between the proportion of boys and girls meeting each of the guidelines were assessed using chi-squared tests and given identified differences all further analyses were stratified by sex. Logistic regression models were used to assess the odds of meeting the separate guidelines by infant and maternal characteristics (i.e., infant temperamental ease, hours per week infant is cared for by someone other than his/her parents, and maternal education). Odds of meeting the combined guidelines were not examined by infant and maternal characteristics due to the low proportion meeting the combined guidelines. Regression analyses were stratified by infant sex and adjusted for infant age and clustering by unit of recruitment (first-time parents’ group). All analyses were conducted in Stata Version 14.0 (StataCorp Texas, USA).

## Results

A total of 542 participants (86% response) took part in the Melbourne InFANT Program. The analytic sample for this paper was 455 participants. Infant and family characteristics are presented in Table 1. Briefly, infants had a mean age of 3.6 months (SD = 1.0) and just over half were boys. Mothers had a mean age of 32.6 years (SD = 4.2).

**Table 1:** Infant and maternal characteristics, *n* = 455

Characteristic	Mean (SD) or %
INFANTS	
Age (mo), mean (SD)	3.6 (1.0)
Male (%)	54.1
zBMI, mean (SD)	−0.5 (1.1)
Weight-for-age percentile, mean (SD)	44.0 (30.3)
Temperamental ease (%)	
Easier than average	56.9
Average	36.3
More difficult than average	6.8
Cared for by someone other than parents (h/week), mean (SD)	1.0 (4.1)
MOTHERS	
Age (y), mean (SD)	32.6 (4.2)
BMI before pregnancy, mean (SD)	24.5 (5.3)
Education level (%)	
Low (completed up to final year of secondary school)	20.4
Intermediate (completed trade/certificate post-secondary school)	24.6
High (completed university degree or beyond)	54.9
Born in Australia (%)	79.3
English is main language spoken at home (%)	95.0

Table 2 reports the proportion of boys and girls meeting each of the guidelines. Around 30% of infants overall met the physical activity (tummy time) guideline, and similarly the screen time guideline. Just under 60% of infants met the restraint and sleep guidelines. For the sleep guideline, which contains a range rather than a threshold, 21.5% of those infants who did not meet the guideline were reported to have too much sleep (above the upper limit of the range) and 78.5% were reported to have too little sleep. Few infants met none of the guidelines (*n* = 42; 9.3%). Similarly few met all of the combined guidelines (*n* = 16; 3.5%). The majority of infants met either one (*n* = 150; 33.1%) or two (*n* = 164; 36.2%) of the four guidelines.

**Table 2:** Proportion of infants meeting guidelines, n (%)^a^

Individual guideline met	Total sample	Boys	Girls	Difference
Tummy time	135 (29.7)	66 (26.8)	69 (33.0)	*p* = 0.15
Restraint	259 (56.9)	133 (54.1)	126 (60.3)	*p* = 0.18
Screen time	127 (27.9)	59 (24.0)	68 (32.5)	p = 0.04
Sleep	267 (58.7)	144 (58.5)	123 (58.8)	*p* = 0.95
Number of guidelines met				
No guidelines	42 (9.3)	22 (9.0)	20 (9.5)	*p* = 0.84
1 guideline	150 (33.1)	85 (34.8)	65 (31.1)	*p* = 0.40
2 guidelines	164 (36.2)	101 (41.4)	63 (30.1)	*p* = 0.01
3 guidelines	81 (17.9)	32 (13.1)	49 (23.4)	*p* = 0.004
All (combined) guidelines	16 (3.5)	4 (1.6)	12 (5.7)	*p* = 0.02

^a^Sex differences based on Chi-Square tests

A higher proportion of girls than boys met each of the guidelines; however, this difference was only significant for screen time. A higher proportion of girls than boys also met three (23.4% versus 13.1%, *p* = 0.004) and all four of the combined guidelines (5.7% versus 1.6%, *p* = 0.02), with a lower proportion of girls meeting 2 guidelines (30.1% versus 41.4%, *p* = 0.01) and no sex difference for the proportion meeting one or none of the guidelines. Infant age (range 0.4 to 6.6 months; continuous variable) was positively associated with meeting the physical activity guideline for both boys (OR 1.46, 95% CI 1.08, 1.96) and girls (OR 1.46, 95% CI 1.09, 1.96) and with meeting the sleep guidelines for boys only (OR 1.64, 95% CI 1.22, 2.22). Infant age was inversely associated with meeting the screen time guideline for girls only (OR 0.70, 95% CI 0.52, 0.98).

Few associations were seen between maternal and infant characteristics and meeting each of the guidelines. No associations were observed for infant temperamental ease, or the amount of time the infant is typically cared for by someone else during the week. The only characteristic associated with odds of meeting any of the guidelines was maternal education and for girls only. Girls had significantly higher odds of meeting the screen time guideline if their mother had a high level of education compared to girls whose mother had a low level of education (OR 3.24, 95% CI 1.22, 8.59). Additionally, girls had significantly higher odds of meeting the sleep guideline if their mother had an intermediate (OR 2.44, 95% CI 1.01, 5.88) or high (OR 4.30, 95% CI 1.95, 9.46) level of education compared to girls whose mother had a low level of education.

## Discussion

With the release of new Australian 24-hour Movement Guidelines for the Early Years it is timely to assess how many children meet the guidelines, and provide a baseline for public health monitoring. Little research exists that describes physical activity and sedentary behaviours in infant populations, and even less that compares behaviour prevalence to guidelines. This study is unique in reporting on guideline adherence across a number of behaviours (physical activity, sedentary behaviours and sleep) in a single cohort of infants. Results indicate that a large proportion of infants in this sample did not meet guidelines, suggesting that there is scope for research to help us better understand why guidelines are not being met and a need for public health campaigns for parents and others who care for infants, to promote strategies to improve adherence.

Less than one third of infants met the physical activity guideline to engage in at least 30 min of tummy time throughout the day. This estimate corresponds to those from previous studies of tummy time in the USA [12–14] with two- and four-month old infants. While tummy time was encouraged in the previous Australian, Canadian and UK guidelines, the addition of a time-specific guideline is new. The finding that the majority of infants do not achieve the recommended amount of tummy time suggests the need for strategies to improve this. Tummy time can be challenging for parents and carers as infants often take time to ‘get used to’ and enjoy this activity, as attested to by the huge volume of information available on parenting websites offering advice about how to encourage this activity (e.g. http://raisingchildren.net.au/articles/pip_tummy_time.html; accessed 10 June 2017). Given the important health and developmental benefits of tummy time, including motor development needed to reach developmental milestones, [1] further strategies are required to assist parents and other caregivers in persisting with the encouragement of this behaviour in their infants.

There are two components to the sedentary behaviour guidelines: screen time and time spent restrained (in situations that restrict movement). Both remain unchanged from the previous Australian guidelines. Infants and those under 2 years of age are encouraged to have no screen time. In the current sample less than one third of infants achieved this guideline, at the lower end of the range reported in a recent systematic review [9]. Further, given only television viewing was assessed in this study, it is likely that the true proportion meeting the guideline may be even lower, despite television viewing being the dominant screen exposure in the first 2 years of life [9], although this may be changing with the development infiltration of technology since this study was undertaken. A higher proportion of girls than boys met the guideline which is at odds with prior evidence indicating no difference in screen time between boys and girls in the under 3 age group, [24] although studies in that review were mostly at the older end of the age spectrum. Thus it is unclear whether this is a spurious finding or whether in this very young population there is a sex difference in screen prevalence. If there is a true sex difference it is not clear what would explain that but in this study it does not appear to be associated with child temperamental ease, or a perception that boys may be “more difficult than average” (and hence placated with screen time as observed in other studies [25]). Clearly strategies from early life to ensure limits are placed on children’s screen time are warranted and specific strategies targeting parents and carers of boys may be required to stem the reliance on screen time for boys.

The guidelines suggest infants and young children should not be kept restrained for more than 1 hour at a time. Restraint is an aspect of sedentary behaviour that has received little attention in the literature [9]. This is the first study to assess compliance with this guideline and indicated that more than half of infants meet it, more than twice as many as meet the screen time guideline. Given the range of situations encapsulated under the term restraint, the measurement of this guideline is difficult to operationalise. In this study restraint was defined as no more than 1 hour per day in any one situation that restricts movement. It is possible that this operationalisation may underestimate adherence, as the time reported in each type of restraint across the day may not have been accumulated in a single session. Conversely, it is possible that this operationalisation may overestimate adherence if infants are experiencing consecutive periods of restraint in different situations e.g. 20 min in the stroller then transferred to the car seat for 30 min and then immediately back into the stroller for 20 min. Further research is needed to better understand this type of sedentary behaviour and how it is accumulated across the day to inform both measurement of compliance and compliance itself.

Sufficient sleep is an integral requirement for growth and development [26, 27] and is a new component included in the movement guidelines. In this study, around 60% of infants met the guidelines appropriate for their age. Interestingly approximately one quarter of those who did not meet guidelines were reported to spend more time sleeping than recommended, with the remainder reported to sleep less than suggested in the guidelines. Good sleep hygiene, particularly regular bedtime routines, are crucial to quality sleep time [27]. While sleep quality was not explicitly assessed in this study, the finding of an association between sleep and maternal education, with infants of mothers with low levels of education having significantly less sleep than infants with mothers who have attained intermediate or high levels of education, may be a reflection of poor sleep hygiene. A study in 3 year olds found less educated mothers employed sleep hygiene bedtime routines less than their more educated counterparts, as did those in low income households [28]. The authors of that study speculated that this association with family disadvantage may reflect less awareness of the importance of sleep hygiene or lower levels of family structure and routines in general due to increased household stress [28]. Given the range of adverse outcomes children in low socioeconomic families are at increased risk of, this association with poorer sleep represents yet another factor which may contribute to disadvantage and suggests the need for early intervention.

Less than 4%, merely 16 infants in this cohort, met the combined 24-hour Movement Guidelines for the Early Years. This indicates that rather than a dichotomy of some infants consistently achieving recommended thresholds and others not, in most cases infants are doing well for some behaviours and require improvement in respect to other behaviours. Girls were more likely to meet all of the guidelines than boys, reflecting the observed trend of higher compliance amongst girls for each individual guideline. There is a need for strategies to assist parents and caregivers in facilitating their infants’ positive behavioural development from the first months of life, particularly for boys.

A strength of this study was the novel investigation of infants in the first few months of life. However, the young age of the participants precluded the investigation of cross sectional associations with potential outcomes given insufficient time for behaviours to have had an impact on outcomes, suggesting a need for longitudinal research in this area. A limitation of this study was the subjective reporting of infant behaviours which may be subject to bias. Further, some of the items demonstrated low test-retest reliability. It is unclear whether this is a reflection of poor measurement or true variability in these behaviours. Other studies have also speculated that young children’s activity behaviours may vary from day to day and week to week [21]. The dearth of instruments for use in this population warrants further attention. The breadth of behaviours captured within a single cohort, and ability to assess adherence with all of the guidelines were a major strength. However, there were limitations with the ability of these pre-collected measures to precisely capture the behaviours of interest, with regard to sedentary behaviour and sleep. While the guidelines specify not being kept restrained for more than 1 hour at a time, in this study restraint was assessed individually for a variety of forms of restraint. Therefore, consecutive time spent restrained was not able to be determined. The guidelines specify amount of time spent in good quality sleep; however, what constitutes good quality is not specifically defined. In this study sleep duration was assessed but quality of sleep was not incorporated into this assessment. Even if items were to be purpose designed to assess the new guidelines, the operationalisation of these aspects of the guidelines is likely to be difficult.

The large sample size and high response rate was a strength of this study. The high proportion of university educated mothers in the sample was despite recruitment across a range of socioeconomic areas [20]. The sociodemographic profile is comparable to the limited data available on first time mothers in Victoria, Australia, as we have reported previously [29]. However, it is acknowledged that this sample may not be representative of all Australian infants. It is likely that the prevalence of meeting guidelines would be lower in a sample containing a higher proportion of low socioeconomic families and multiple child families. In this study infants of low educated mothers were less likely to meet guidelines. Further, it could be hypothesised that parents with multiple children would have greater parenting demands and less time and thus their infants may be less likely to adhere to guidelines.

## Conclusion

In this population of 4 month old infants the vast majority failed to meet all of the guidelines contained in the new Australian 24-hour Movement Guidelines for the Early Years, with most infants meeting only one or two of the individual guidelines and compliance lower amongst boys than girls. This suggests the need for a concerted effort to raise awareness of the importance of these movement behaviours and to provide strategies and support for those providing care for infants, particularly among parents with lower levels of education, to assist them in adhering to the guidelines. A lower proportion of infants met the tummy time and screen time guidelines, compared with the restraint and sleep guidelines, suggesting the greatest need for support is in regard to these behaviours. The release of new guidelines will assist in efforts to optimise movement behaviours in infants by providing a platform for those working with families of young children from which unified advice and support can be provided.

## Abbreviations

BMI: Body mass index
CI: Confidence interval
ICC: Intra-class correlation
OR: Odds ratio
SD: Standard deviation
UK: United Kingdom
USA: United States of America
